# Childhood Hepatitis in Osh Province of Southern Kyrgyzstan

**DOI:** 10.5005/jp-journals-10018-1116

**Published:** 2014-07-28

**Authors:** Rakhmanbek Toichuev, Elena Leybman, Cholpon Omorbekova, Gulbarchyn Rakhimova, Baktygul Zhumabek Kyzy, Lyudmila Nikolaeva

**Affiliations:** 1 Institute of Medical Problems, Southern Branch of the National Academy of Sciences of the Kyrgyz Republic, Osh, Kyrgyzstan; 2 DI Ivanovsky Institute of Virology, Ministry of Healthcare of the Russian Federation, Moscow, Russia; 3 NI Pirogov Medical University, Ministry of Healthcare of the Russian Federation, Moscow, Russia; 4 Osh Interdistrict Children Hospital, Ministry of Healthcare of the Kyrgyz Republic, Osh, Kyrgyzstan

**Keywords:** Children, Hepatitis, Osh Province, Kyrgyzstan.

## Abstract

**Abbreviations:** CHC: Chronic hepatitis C; HCV: Hepatitis C virus; LC: Liver cirrhosis.

**How to cite this article:** Toichuev R, Leybman E, Omorbekova C, Rakhimova G, Zhumabek Kyzy B, Nikolaeva L. Childhood Hepatitis in Osh Province of Southern Kyrgyzstan. Euroasian J Hepato-Gastroenterol 2014;4(2):117-118.

To the Editor,

The Kyrgyz Republic is a high-risk area for viral hepatitis. In Kyrgyzstan, during 2000 to 2008, acute viral hepatitis had the following etiological pattern: hepatitis A—61%, hepatitis B — 17%, hepatitis C—4.5%, hepatitis D — 1.7%, mixed hepatitis (B + C) — 15%.^J^ The distribution pattern of etiological factors has not significantly changed till now. Up to 85 to 90% of the patients with hepatitis A were children, in whom 0.7% were younger than 1 year old, 5% were 1 to 2 years old, 42.7% were 2 to 5 years old, 51.6% were 6 to 14 years old.^1,2^ The most susceptible areas for enteral viral hepatitis include Osh, Jalal-Abad and Naryn provinces, where 80% of children display antibody to hepatitis A virus and 8.3% of the entire population show antibody to hepatitis E virus.^2^ During recent years, an increase in the incidence of the hepatitis C was noticed in Kyrgyzstan.^3^ The official registration of hepatitis C was started in 1996. About 3.6% of all patients with chronic hepatitis C (CHC) are children.^3^ Hepatitis C virus (HCV) genotypes 1 (56.7%), 3 (23.1%), and 2 (8.6%) are predominated in Kyrgyzstan.^4^

The most serious situation with hepatitis is in Osh province (southern area of Kyrgyzstan).^5^ Hepatitis is diagnosed about 5 to 6 times more in Osh province than in the other provinces of the Northern region of Kyr-gyzstan.^5^ A considerable part of Osh population work at cotton and tobacco growing fields, where organo-chlorine pesticides (hexachlorocyclohexane or lindane, dichlorodiphenyltrichloroethane, aldrin, and dieldrin) are used. The pesticides can be ingested by people together with food and water. The etiologic factors of childhood hepatitis in Osh Inter-district Children Hospital during 2011 to 2013 were evaluated. Children with different hepatitis, except viral hepatitis A and E, are treated in this hospital. Four hundred and twenty-eight children (2-14 years old) with clinical presentation of hepatitis were analyzed. The levels of alanine and aspartate aminotransferases, bilirubin (total, direct and indirect), thymol index, serum protein, albumin, and globulins were detected in sera using Screen Master (’Hospitex Diagnostics’, Florence, Italy). The etiologic factors of hepatitis were evaluated as it was recommended in the International Classification of Diseases.^6^ Viral hepatitis B and C were diagnosed in according with detection of the viral DNA or RNA, specific antibody, and/or hepatitis B surface antigen. Diagnoses of chronic hepatitis B or C were done according to the standard classification. Cases of acute hepatitis B and C were not assessed in this study. Hepatitis lesions caused by cytomegalovirus infection was confirmed, if immunoglobulin M specific to the virus and its DNA were detected. Hepatitis, caused by parasitic infection, was diagnosed, if eggs of *Fasciola hepatica* were discovered in fecal samples or in bile. Drug-induced hepatitis was confirmed, if patient received antituberculosis, antiepilepsy and anti-inflammation drugs and showed lasting changes in liver biochemistry. Toxic liver disease with hepatitis symptoms was diagnosed, if patient had pesticides in blood. Non-specific reactive hepatitis was diagnosed, if patient displayed changes in liver biochemistry and was infected by bacteria (mainly by *Brucella melitensis* or *Salmonella enterica* serotype typhi) and by virus (mainly by human herpes virus type IV or VI). The etiologic structure of hepatitis incidences in children of the Osh Inter-district Children Hospital is presented in Table 1. The following remarkable trait was the prevalence of chronic viral hepatitis B and C (together about 32.2% of all cases). The share of toxic (pesticide-induce) liver disease with hepatitis symptoms was 6.5%, which is obviously a significant finding. Scarcity of publications on toxic pesticide-induced hepatitis in children in the literature should be pointed out. A level of the pesticides in blood tended to be higher by the end of the agricultural works. According to the data of liver biopsy of both adults and children, who had died in accidents in Osh province, the pesticides were detected in the liver.^5^ In this communication, the share of cryptogenous hepatitis (32.7%) was overstated. There may be various reasons for this. Undiagnosed autoimmune hepatitis and viral hepatitis caused by TT virus, adenovirus and by other agents may be underlying factor. Also, the sensitivity and specificity of different assay systems need to be explored in future. Liver cirrhosis (LC) was detected in 12 children (2.8% of all cases). Around 16.6% of all cirrhosis cases were of HCV etiology, which is higher than in Russian children from Moscow (4.3%).^7^ According to the data reported by different researchers, 1 to 10% of children with CHC developed cirrhosis.^8,9^ In our communication, children with CHC-LC showed different blood biochemistry, when compared to children with CHC without cirrhosis. Differences were detected in the values of bilirubin, cholesterol, p-lipoproteins, y-globulin and thymol index (data not shown). The similar data for Russian children with CHC-LC *vs* CHC were published previously.^10^ In conclusion, further studies of epidemiological situation and ecologic factor influencing childhood hepatitis in Osh province should be studied in details. Different preventive measures should be implemented. As LC has the greatest socioeconomical impact, the risk of cirrhosis development must be determined and preventive measures should be adopted in children.

**Table 1:** **Table 1:** The etiologic factors of childhood hepatitis in Osh Inter-district Children Hospital

					*Percentage* * (numbers)*	
I.		Chronic viral hepatitis:				
		• Hepatitis B			18.2% (78)	
		• Hepatitis C			14% (60)	
II.		Hepatic lesions in infectious and parasitic diseases:				
		• Cytomegalovirus infection			2.8% (12)	
		• Parasitic infection			2.8% (12)	
III.		Nonspecific reactive hepatitis			19.2% (82)	
IV.		Toxic liver disease:				
		• Medicine-induced liver disease			3.7% (16)	
		• Toxic liver disease with hepatitis symptoms (toxic hepatitis)			6.5% (28)	
V.		Cryptogenic hepatitis			32.7% (140)	
